# Challenges in the Management of Type 2 Diabetes Mellitus and Cardiovascular Risk Factors in Obese Subjects: What Is the Evidence and What Are the Myths?

**DOI:** 10.1155/2013/856793

**Published:** 2013-06-10

**Authors:** Lovely Chhabra, Besiana Liti, Gayatri Kuraganti, Sudesh Kaul, Nitin Trivedi

**Affiliations:** ^1^Department of Internal Medicine, Saint Vincent Hospital, University of Massachusetts Medical School, Worcester, MA 01608, USA; ^2^Department of Endocrinology, Saint Vincent Hospital, University of Massachusetts Medical School, Worcester, MA 01608, USA

## Abstract

The increasing worldwide prevalence of diabetes mellitus and obesity has projected concerns for increasing burden of cardiovascular morbidity and mortality. The dangers of obesity in adults and children have received more attention than ever in the recent years as more research data becomes available regarding the long-term health outcomes. Weight loss in obese and overweight subjects can be induced via intensive lifestyle modifications, medications, and/or bariatric surgery. These methods have been shown to confer overall health benefits; however, their effect on remission of preexisting diabetes mellitus and reduction in cardiovascular risk has been variable. Recent research data has offered a much better understanding of the pathophysiology and outcomes of these management strategies in obese patients. In this paper, the authors have summarized the results of major studies on remission of type 2 diabetes mellitus and reduction of cardiovascular events by weight loss induced by different methods. Furthermore, the paper aims to clarify various prevailing myths and practice patterns about obesity management among clinicians.

## 1. Introduction

Historically, among various cultures, weight gain has been viewed as a sign of wealth and prosperity but as the dynamics of food production and consumption have changed, the world now faces an epidemic of obesity. According to the World Health Organization (WHO), worldwide prevalence of obesity has doubled since 1980 with estimated 1.5 billion adults with obesity in 2008 [1]. In the United States, more than 64% of the population is overweight (BMI ≥ 25 kg/m^2^), and more than 33% of the adult population meets criteria for obesity (BMI ≥ 30 kg/m^2^) [2]. On a similar note, more than 25 million US adults have type 2 diabetes mellitus, and this figure will likely reach 50 million by 2050 given the current demographic trends and continued progression of obesity [3, 4]. The increasing worldwide prevalence of diabetes mellitus and obesity has projected concerns for increasing burden of cardiovascular morbidity and mortality. The dangers of obesity in adults and children have received more attention than ever in the recent years as more research data becomes available regarding the long-term health outcomes. The increasing rates of diabetes in children and adolescents and the limited capacity of the current therapeutic treatments to slow the disease progression raise the concern for a full blown diabetes tsunami for the generations to come [5]. Weight loss in obese and overweight subjects can be induced via intensive lifestyle modifications, medications, and/or bariatric surgery. These methods have been shown to confer overall health benefits; however, their effect on remission of preexisting diabetes mellitus and reduction in cardiovascular risk has been variable. Recent research data has offered a much better understanding of the pathophysiology and outcomes of these management strategies in obese patients. In this paper, the authors have summarized the results of major studies on remission of type 2 diabetes mellitus and reduction of cardiovascular events by weight loss induced by different methods. Furthermore, the paper aims to clarify various prevailing myths and practice patterns about obesity management among clinicians.

## 2. Methods

In preparation for this paper, several online search engines were used to gather journal articles that focused on studying the efficacy of surgical weight loss as compared to the conventional medical therapies and intensive lifestyle modifications. An initial advanced literature search was conducted by PubMed and MEDLINE using a combination of key words that included “epidemiology,” “obesity,” “weight loss,” “diabetes mellitus,” and “bariatric surgery” which yielded 831 articles. The search was then narrowed to articles published in the last two decades. Furthermore, an individual screening of the articles was conducted for prospective, randomized, and controlled trials comparing the effectiveness of surgical versus medical/lifestyle intervention for weight loss and long-term cardiovascular and diabetes outcomes. Also, the Cochrane collaborative database was utilized to obtain articles on the latest information on weight loss and its effects on cardiovascular risk. To write our paper, we finally selected 56 articles, based on the study design and power, which were relevant to our current discussion. Studies referred were original research, both prospective and retrospective controlled studies, and three large meta-analyses. The current review aims to draw evidence based conclusions, utilizing the latest research data, which can be utilized to guide the treatment of the obese diabetic patient population. 

## 3. Data and Discussion

Recent data has suggested that diabetes mellitus may be reversed or prevented with weight loss strategies in obesity [4, 6]. The exact definition of diabetes remission remains an area of debate [7]; however, the American Diabetes Association (ADA) currently defines this as the achievement of euglycemia without pharmacological treatment for at least 1 year [8]. Partial diabetes remission is defined as a transition from meeting diabetes criteria to a prediabetes glycemia level (i.e., fasting plasma glucose of 100–126 mg/dL and HbA1c of 5.7–6.5%) and complete diabetes remission as the transition from diabetes criteria to full glucose normalization (i.e., fasting plasma glucose < 100 mg/dL and HbA1c < 5.7%) [9]. Although this is a good operational definition, according to glycemic and pharmacological criteria, it does not account the underlying beta-cell function and insulin action and thus may be inappropriate to define a cure of diabetes [10]. 

The most recently published data from the Look AHEAD trial showed that the intensive lifestyle intervention (ILI) is superior to conventional diabetes education in inducing weight loss and partial or complete remission of diabetes mellitus [9]. ILI included weekly group and individual counseling in the first 6 months, followed by 3 sessions per month for the second 6 months and twice-monthly contact and regular refresher group series and campaigns for 3 years. The ILI aimed to limit total caloric intake to 1200 to 1800 kcal/d through reductions in total and saturated fat intake and by increasing physical activity levels to a goal of 175 min/wk. Liquid meal replacements were provided to assist dietary goals. Participants in the conventional diabetes education were offered 3 group sessions each year focusing on diet, physical activity, and social support. At 2, 3, and 4 years, respectively, 9.2%, 6.4%, and 3.5% of intensive lifestyle intervention participants (*n* = 2262) had partial diabetes remission compared with 1.7%, 1.3%, and 0.5% of participants in the diabetes support and education group (*n* = 2241). Complete remission was; however, rare: 1.3% and 0.7% at 1 and 4 years. Participants with early stage diabetes (shortest duration, not treated with insulin, good baseline glycemic control) were the most to benefit from ILI. Furthermore, the study also showed that ILI leads to significant improvements in other health indicators, such as body weight, fitness, blood pressure, glycemic control, and lipids. Despite improvements in risk factor profiles, ILI did not result in decrease of cardiovascular events (nonfatal myocardial infarction, hospitalization for angina, nonfatal stroke, or death) compared to conventional diabetes education and therapy. As the primary end point was not met, it led to premature termination of the Look AHEAD trial. The results of the Look AHEAD trial are somewhat similar to Action in Diabetes and Vascular Disease (ADVANCE) trial, Action to Control Cardiovascular Disease in Diabetes (ACCORD) trial, and Veterans Affairs Diabetes Trial (VADT). In these trials, intensive medical/antiglycemic therapy did not show any cardiovascular benefit despite better diabetes control and improvement in cardiovascular risk factors. In the ACCORD trial, the mortality rate was about 19% higher in the intensive glucose management group as compared to standard glucose management group [11–13]. Nevertheless, it should be noted that most patients in the ACCORD, ADVANCE, and VADT trials had longstanding diabetes with preexisting overt cardiovascular disease. In contrast to these studies, intensive antiglycemic therapy in newly diagnosed type 2 diabetics (UKPDS study) without overt coronary heart disease resulted in reduction in coronary events. Nonetheless, the effect on cardiovascular risk reduction became apparent after several years of treatment [14]. One may argue that the results of the look AHEAD trial could be similar to the UKPDS study with longer followup, but the results could have been affected by the use of statins and medical therapy including angiotensin converting enzyme inhibitors in both the study groups.

Medical therapy for obesity with orlistat and sibutramine results in modest weight loss. Orlistat leads to about 3 kg weight loss; however, no reduction in cardiovascular events has been shown despite the weight loss and improvement in diabetes mellitus and lipid parameters [15]. Sibutramine use has been linked to increased number of nonfatal myocardial infarction and stroke despite weight loss [14], and for this reason, sibutramine was withdrawn from the market in October 2010. 

Two other studies, the UKPDS and STENO trials, reported important findings in regards to reduction in cardiovascular events with better diabetes control. The open, parallel STENO trial studied a composite end point of death from cardiovascular causes, nonfatal myocardial infarction, nonfatal stroke, revascularization and amputation in patients with type 2 diabetes. The study concluded that a focused multifactorial intervention that includes pharmacological therapy along with behavior modification and aspirin therapy decreased the risk of cardiovascular disease and the overall levels of glycosylated hemoglobin, blood pressure, cholesterol, and triglycerides levels as well as urinary albumin excretion [16]. Interestingly, the 13-year followup to the STENO trial (STENO-2) did not reveal any significant weight loss in either study groups despite the sustained reduction in cardiovascular events in the intensive therapy group posing a question for whether weight loss plays a key role in cardiovascular risk reduction [17]. The study showed a 50% overall risk reduction in cardiovascular and microvascular events with the implementation of the intensive multifactorial treatment [16]. The UKPDS trial on the other hand, randomly assigned 4209 patients with newly diagnosed type 2 diabetes to either conventional therapy (dietary restriction) or intensive therapy (sulfonylurea or insulin or in obese subjects metformin). The study's 10-year followup concluded that despite the loss of glycemic differences between the two groups, a continued reduction in microvascular risk and an overall risk reduction for myocardial infarction and death from any cause were observed [18]. A summary of the main findings of various research studies is enclosed in Table 1, whereas Table 2 gives a summary of the baseline characteristics of the study participants for the ACCORD, UKPDS, Look AHEAD, VADT, and ADVANCE trials. 

There is increasing evidence that bariatric surgery is more effective than medical or lifestyle interventions for weight loss, reduction in cardiovascular risk, and diabetes remission. The reduction in comorbidities by bariatric surgery appears to translate into a 29% mortality reduction rate [19, 20]. A large cohort study of nearly 8000 patients who had undergone Roux-en-Y surgery was matched to a similar sized cohort. In this study the patients with Roux-en-Y had a reduction of all-cause mortality by 40%, diabetes-related mortality by 92%, coronary artery disease related mortality by 56%, and mortality from cancers by 60% [21]. A large meta-analysis published in 2009 based on the results of 621 studies (from 1990 to 2006) reported that average weight loss by a bariatric surgery was about 38.5 kg or 55.9% excess body weight loss. Overall, 78.1% of diabetes patients had complete resolution and diabetes was improved or resolved in about 86.6% of the patients. Weight loss and diabetes resolution were found to be the greatest for patients undergoing biliopancreatic diversion/duodenal switch, followed by gastric bypass, and the least with banding procedure [22]. In a recent analysis of severely obese patients with diabetes, the adjusted probability of initial remission of diabetes mellitus was found to be 12-to-24-fold greater for patients who underwent bariatric surgery than for those who underwent medical care alone. Bariatric subjects also experienced lower relapse rates of diabetes compared to the nonsurgical subjects and without higher risk of death [23]. 

Another large multisite retrospective cohort study of adults with uncontrolled or medication-controlled type 2 diabetes who underwent gastric bypass reported that 68% patients experienced an initial complete diabetes remission within 5 years after surgery, of which 35% redeveloped diabetes within 5 years. Significant negative predictors of diabetes remission were poor preoperative glycemic control, insulin use, and longer diabetes duration. Weight trajectories after surgery were significantly different for never remitters, relapsers, and durable remitters [24]. Furthermore, Adams et al. recently reported the results of a prospective study which gathered data 6 years after Roux-en-Y gastric bypass (RYGB) surgery and aimed to examine the association of the surgery with weight loss, diabetes mellitus, and other health risks. They found that remission of type 2 diabetes was significantly more common among patients who underwent RYGB (62%) than among severely obese subjects with diabetes who initially sought but did not undergo bariatric surgery (6%) and those who never sought bariatric surgery (8%) [25]. 

The Swedish Obese Subjects (SOS) study is an ongoing, nonrandomized, controlled, prospective, and matched study conducted at 25 public surgical departments and 480 primary health care centers in Sweden of 2010 obese participants who underwent bariatric surgery and 2037 contemporaneously matched obese controls who received usual care. Sjöström et al. published the 2-year and 10-year follow-up results of SOS study in 2004 where they found that after two years, the weight had increased by 0.1 percent in the control group and had decreased by 23.4 percent in the surgery group (*P* < 0.001) and after 10 years, the weight had increased by 1.6 percent in the control group and decreased by 16.1 percent in the surgery group (*P* < 0.001). Energy intake was lower and the proportion of physically active subjects higher in the surgery group than in the control group throughout the observation period. Two- and 10-year rates of recovery from diabetes, hypertriglyceridemia, low levels of high-density lipoprotein cholesterol, hypertension, and hyperuricemia were more favorable in the surgery group than in the control group, whereas recovery from hypercholesterolemia did not differ between the groups. The surgery group had lower 2- and 10-year incidence rates of diabetes, hypertriglyceridemia, and hyperuricemia than those of the control group; differences between the groups in the incidence of hypercholesterolemia and hypertension were; however, undetectable [26]. Sjöström et al. published another followup results of the SOS study in 2012 where they reported that bariatric surgery was associated with reduced incidence of fatal and total cardiovascular events, myocardial infarction and stroke even after the adjustment of baseline conditions (adjusted hazard ratio of 0.47; 95% CI 0.29–0.76; *P* = 0.002 for all cardiovascular deaths and adjusted hazard ratio of 0.67; 95% CI 0.54–0.83; *P* < 0.001 for all first time fatal or nonfatal cardiovascular events) [27]. 

Another prospective, nonrandomized, and controlled one year clinical trial aimed to compare the effects of Roux-en-Y gastric bypass surgery and comprehensive lifestyle intervention on type 2 diabetes and obesity-related cardiovascular risk factors reported mean one-year weight loss of 30% and 8% respectively in the surgical and medical intervention groups [28]. Beneficial effects on glucose metabolism, blood pressure, lipids, and low-grade inflammation were observed in both groups. Remission rates of type 2 diabetes and hypertension were significantly higher in the surgery group than the lifestyle intervention group; 70 versus 33%, *P* = 0.027, and 49 versus 23%, *P* = 0.016. The surgery group experienced a significantly greater reduction in the prevalence of metabolic syndrome, albuminuria, and electrocardiographic left ventricular hypertrophy than the lifestyle group. Gastrointestinal symptoms and symptomatic postprandial hypoglycemia developed more frequently after gastric bypass surgery than after lifestyle intervention, though mortality in either group was not significantly different [28]. One very recent prospective, randomized, and nonblinded clinical trial with one-year follow-up period, performed at the Cleveland Clinic, evaluated the efficacy of intensive medical therapy alone versus medical therapy combined with Roux-en-Y gastric bypass or sleeve gastrectomy in 150 obese patients with uncontrolled diabetes mellitus and reported HbA1c reduction to ≤6% in 12% patients receiving medical therapy versus 42% in gastric-bypass group and 37% in sleeve-gastrectomy group [29]. Weight loss and improvement in insulin resistance was significantly higher in both the surgical groups than in medical therapy only group. The use of drugs to lower glucose, lipid, and blood-pressure levels decreased significantly after both surgical procedures but increased in patients receiving medical therapy only [29]. Mingrone et al. conducted a single-center, nonblinded, randomized, and controlled trial comparing the efficacy of Roux-en-Y gastric bypass and biliopancreatic diversion versus medical therapy for the remission of diabetes mellitus in morbidly obese diabetic patients. At 2 years, diabetes remission occurred in no patients in the medical-therapy group versus 75% in the gastric-bypass group and 95% in the biliopancreatic-diversion group (*P* < 0.001). Age, sex, baseline BMI, duration of diabetes, and weight changes were not significant predictors of diabetes remission or of improvement in glycemia. Though the average baseline glycated hemoglobin level had decreased in all groups, patients in both the surgical groups had greater degree of glycemia improvement than those of medical therapy group, the greatest improvement to have noted in the biliopancreatic-diversion group [30]. 

Though previous studies have offered a strong evidence of benefit of bariatric surgery in patients with a BMI >40 kg/m^2^, the benefits in patients with a lower BMI obese patients, that is, mild (BMI = 30–35 kg/m^2^) or moderate obesity (BMI = 35–40 kg/m^2^), have remained unclear until recently [31]. Current indications of bariatric surgery in obese patients as defined by the National Institute of Health are: BMI > 40 kg/m^2^ OR BMI > 35 kg/m^2^ associated with serious comorbidities like diabetes, sleep apnea, obesity-related cardiomyopathy, or severe joint disease [32]. However, recent data also suggests benefit of bariatric surgery in mild and moderate obesity patients. A recent prospective study compared the effects of laparoscopic sleeve gastrectomy (LSG) versus medical therapy on patients with type 2 DM and a BMI of <35 kg/m^2^ and reported diabetes mellitus and hypertension remission rate of 88.8% (8 of 9 patients) without undesirable excessive weight loss as compared to none of the patients (0 of 9 patients) undergoing remission in the medical therapy group [33]. Another prospective, consecutive, and nonrandomized trial of 79 patients investigate the role of sleeve gastrectomy for patients with class I obesity (BMI 30–35 kg/m^2^) and reported promising early weight loss and quality of life improvement in patients [34]. One of the largest, prospective randomized trials published to date studying the long-term impacts of RYGB (followup being 6 years) on patients with diabetes and only class I obesity reported durable diabetes remission occurred in 88% of cases, with glycemic improvement in 11% [35]. Mean HbA1c fell from 9.7 ± 1.5 to 5.9 ± 0.1% (*P* < 0.001), despite diabetes medication cessation in the majority. Weight loss failed to correlate with several measures of improved glucose homeostasis, consistent with weight-independent antidiabetes mechanisms of RYGB. C-peptide responses to glucose increased substantially, suggesting improved *β*-cell function. There was no mortality, major surgical morbidity, or excessive weight loss. Hypertension and dyslipidemia also improved, yielding 50–84% reductions in predicted 10-year cardiovascular disease risks of fatal and nonfatal coronary heart disease and stroke [35]. In addition, post hoc study findings reported by Sjöström et al. demonstrated that a higher baseline BMI may not be associated with a greater benefit of bariatric surgery [27].

Other important comorbidities that deserve to be mentioned in this paper are nonalcoholic fatty liver disease (NAFLD) and non-alcoholic steatohepatitis (NASH) as they may frequently be considered a part of the metabolic syndrome (insulin resistance). Some studies have reported improvement in NAFLD, adipokines (used to measure fatty liver disease), and insulin resistance after weight loss with bariatric surgery [36, 37]. However, randomized trials to confirm these findings are lacking and thus use of surgical approach for these conditions remains controversial [38].

Though our current discussion mainly focuses on the review and comparison of effectiveness of medical versus surgical weight loss strategies; however, we wanted to mention here the results of another most recent randomized trial which showed that the use of Mediterranean diet results in the reduction of major cardiovascular events especially in obese patient population [39]. This study mainly compared the cardiovascular outcomes in patients receiving Mediterranean diet versus those receiving regular controlled diet. None of the study patients were advised either total calorie restriction or promotion of physical activity. The effects of Mediterranean diet on the weight/BMI were also not the major focus of this study. To best of our knowledge, this study is the first largest prospective study which showed significant cardiovascular risk reduction in obese patients with the use of Mediterranean diet and thus this opens up a consideration for recommending the use of this dietary lifestyle intervention which may potentially reduce cardiovascular risk in obese patients.

## 4. Summary and Future Directions 

Look AHEAD trial, the largest prospective trial comparing efficacy of ILI with standard diabetes education and management (SDE) in obese diabetics, failed to show any additive benefit of ILI over SDE in the improvement of cardiovascular outcomes despite an observed improvement in weight loss, body fitness, and diabetes remission. Complete diabetes remission with ILI was rare, but partial diabetes remission even though significant in the initial years was not long lasting. Partial diabetes remission with ILI was most effectively achieved in patients with short duration diabetes history, who have lower HbA1c levels and those who do not yet require insulin therapy, suggesting that emphasis on early diabetes screening and probably intensive/aggressive management in the early course of the disease warrants more attention. Some prior studies of medication based therapy have shown that reductions in HbA1c and fasting glucose, if sustained, are likely to considerably reduce the risks of microvascular complications [40, 41]. One could argue that failure of demonstrating any benefit in the cardiovascular outcomes with ILI in the Look AHEAD trial could be because of improved cardiovascular risk factor control in both the study groups through medical treatment, improved clinical care after acute cardiovascular events, enrollment of a healthier-than-expected patient cohort and excluding patients with high baseline cardiovascular risk factors [10]. This may need to be confirmed with future long-term prospective studies. However, based on the current evidence, intensive medical therapy alone does not appear to be very promising in improving cardiovascular outcomes or producing long-term diabetes remission.

In contrast to weight loss by medications or intensive lifestyle modification, bariatric surgery has shown to be very effective in improving cardiovascular health and in producing diabetes remission in obese diabetic patients by the majority of both long-term and short-term cohort and prospective studies. Although one may assume that the degree of postoperative weight loss might be the major driver for improvement in diabetes mellitus in obese patients undergoing bariatric surgery, the weight loss and type 2 diabetes are often not in a direct cause-and-effect relationship. The improvement of diabetes mellitus occurs within days after gastric bypass, before there is significant weight loss [42–44]. These findings suggest that bariatric surgeries may affect the diabetes course independent of the weight. Rubino et al. suggested that changes in the gut hormonal milieu after bypass of the distal stomach, duodenum, and proximal jejunum can influence the mechanism of type 2 diabetes [45]. Other mechanisms involved may be changes in neurohumoral regulation, increased expression of glucose transporter type 4 (GLUT4) [46], and normalization of whole-body insulin resistance [30]. 

Though BMI is still the most widely used criteria for selection of patients for bariatric surgery in clinical practice, this practice pattern may change as the recent data shows significant benefit of bariatric surgery in mild and moderately obese patients. Also, one study findings suggested that high insulin levels may be a better selection criterion for bariatric surgery than high BMI, as far as cardiovascular events are concerned [27]. As seen with intensive lifestyle treatment, patients with early stage diabetes appeared to benefit most from bariatric surgery; so a strong argument can be made for considering earlier surgical intervention in moderately obese patients (body mass index ≥ 30) with early stage diabetes. At the same time, offering bariatric surgery to every obese diabetic individual may impose serious practical concerns which include nonbenign nature of the surgical intervention (including some associated long-term mechanical and nutritional complications), nonsustained beneficial long-term effects, as shown by Holman et al. [18] and Arterburn et al. [24], and significant increase in the nationwide healthcare burden related to the cost of intervention. 

## 5. Common Myths about Obesity among Clinicians and Patients

1. *I should advise my obese diabetic patients to follow intensive dietary and exercise (lifestyle) interventions as this would decrease their cardiovascular risk as compared to routine interventions and medical therapy*.
2. *Answer. *This is the most common myth which prevails among clinicians, may it be general internists or cardiologists. Though this statement sounds very conceptual, the prospective studies have jolted this prevailing belief as intensive lifestyle interventions have not shown to decrease cardiovascular morbidity and mortality in these patients despite improvement in other health indicators like weight loss, body fitness and diabetes remission.
3. Though with that being said, data in these studies have their own limitations as discussed earlier and the authors feel it should not preclude clinicians from advising their patients to adopt healthier lifestyle measures including intensive lifestyle interventions for management of diabetes in obesity.
4. *Poorly controlled diabetics with a long standing history of disease are most likely to benefit from intensive lifestyle therapy for their diabetes control or remission. *
5. *Answer. *This is another common myth. As we discussed earlier, data from prospective studies have shown that newly diagnosed diabetics or diabetics with better glycemic control are in fact more likely to achieve a partial or transient complete diabetes remission with intensive lifestyle modification and medical therapy, as compared to patients with long standing history of disease.
6. *Bariatric surgery is more effective than medical management in inducing diabetes remission because it induces rapid and more weight loss. *
7. *Answer. *The improvement of diabetes mellitus occurs within days after gastric bypass, before there is significant weight loss. Changes in the gut hormonal milieu after gastric bypass, changes in neurohumoral regulation, and normalization of whole body insulin resistance have been proposed to be additional mechanisms providing therapeutic benefit.
8. *Weight loss strategies in obesity should include setting of realistic goals because otherwise patients will become frustrated and lose less weight. *
9. *Answer. *Although this sounds a very reasonable concept, several studies have offered contrasting data and support that more ambitious goals are associated with better weight-loss outcomes [47, 48]. Study results were more supportive of the idea that higher goals motivate patients to lose more weight than of the hypothesis that high goals undermine effort.
10. *Large amount and rapid weight loss is associated with poorer long-term outcomes, as compared with slow, gradual weight loss. *
11. *Answer. *This belief probably emerged over five decades ago in reaction to the adverse effects of nutritionally insufficient very-low-calorie diets (<800 kcal per day) and has persisted to the extent that it may be common advise by many regular dieticians. In several prospective trials, patients often undergo more rapid and greater initial weight loss and this has been associated with lower body weight at the end of long-term followup. Some obese persons have a greater initial weight loss than others do due to unclear reasons, thus a recommendation to lose weight more slowly might interfere with the ultimate success of weight-loss efforts [49].
12. *Sexual activity is a good means to burn extra calories. *
13. *Answer. *People often may feel that they spend a lot of energy (about 100–300 kcal) while indulging in one sexual intercourse. It is a myth. Studies have shown that an average bout of sexual activity lasting for about 6 minutes approximately expends only 21 kcal [50].
14. *Breastfeeding is protective against childhood obesity*.
15. *Answer. *Breastfeeding has been advocated to offer a protective benefit against the development of childhood obesity, for a long time. Previously published review report from the World Health Organization also supported this fact [51]. The incidence of type 1 diabetes is reduced by 30% in infants exclusively breastfed for at least 3 months. A reduction of 40% in type 2 diabetes also is reported, possibly reflecting the long-term positive effect of breastfeeding on weight control and feeding self-regulation [52]. However, more recent data from prospective randomized controlled studies do not support the theory of antiobesity effects of breast milk to the children [53–55].
16. *“My obesity is hereditary. Everyone in my family is obese. Dietary or behavioral modifications are not going to work, so why even bother”? *
17. *Answer. *Although genetic factors play a large role in obesity, heritability is not horoscope. Studies have shown that even moderate environmental changes can promote significant weight loss [56]. Identification of key environmental factors and then successful interventions can achieve clinically significant reductions in obesity.
18. *Bariatric surgery offers benefits only in patients with* BMI >40* or* BMI >35* with associated severe comorbidities. *
19. *Answer. *Though BMI is still the most widely used criteria for selection of patients for bariatric surgery in clinical practice, this practice pattern may change as the recent data from multiple studies shows significant benefit of bariatric surgery in mild and moderate obese patients.

## 6. Conclusions

1. The association of diabetes mellitus, obesity, and cardiovascular risk factors is very complex. Recent research data certainly provides a better understanding; however, many pathophysiologic mechanisms associated with the benefits of medical and surgical interventions remain poorly understood. Future long-term prospective studies are warranted for a better clinical decision making.
2. Clinical education, research, and health policies need to increase their focus towards primary prevention of risk factors associated with diabetes mellitus and obesity especially in children and adolescents who are deemed to be at high risk.
3. Though ILI did not appear to reduce the risk of cardiovascular events significantly in prospective studies, the authors feel that it should be recommended to all the patients given the absence of long-term follow-up data and significant health benefits achieved in a subset of patients.
4. Several myths prevail regarding obesity not only among general population but also among clinicians which may affect practice patterns. More education among the patients and general clinicians may produce better healthcare outcomes in obese subjects.
5. Bariatric surgery certainly appears to have an emerging and promising future role in the management of cardiac risk factors and diabetes remission in obese diabetic patients, especially in the subset of the newly diagnosed diabetics in this patient population. Furthermore, bariatric surgical intervention has also shown some promising outcomes in the less severely obese patient population who have high cardiovascular risk factors. However, appropriate selection criteria need a consensus opinion involving possible reformulation of the existing guidelines.

## Figures and Tables

**Table 1:** Summary of the results of the major trials.

Look AHEAD trial	Intensive lifestyle intervention (ILI) is superior to conventional diabetes education in inducing weight loss and partial or complete remission of diabetes mellitus. No benefit of ILI, however, on cardiovascular outcomes was noted

ACCORD trial	Five-year results confirm that neither more intensive lowering of blood glucose levels and more intensive lowering of blood pressure nor treatment of blood lipids with a fibrate and a statin drug reduces cardiovascular risk in people with established type 2 diabetes who are at severely high risk for cardiovascular events. However, the study did find improvements to microvascular conditions. Also, more deaths from any cause and fewer nonfatal myocardial infarctions were observed

VADT	Intensive glucose control in patients with poorly controlled type 2 diabetes had no significant effect on the rates of major cardiovascular events, death, or microvascular complications, with the exception of progression of albuminuria

ADVANCE trial	Intensive medical/antiglycemic therapy did not show any macrovascular benefit despite better diabetes control and improvement in cardiovascular risk factors, but it did show a reduction in microvascular events

UKPDS study	Intensive antiglycemic therapy in newly diagnosed type 2 diabetics without overt coronary heart disease resulted in reduction in coronary events. Study's 10-year follow up concluded that despite the loss of glycemic differences between the two groups, a continued reduction in microvascular risk and an overall risk reduction for myocardial infarction and death from any cause were observed

STENO trial	It concluded that a focused multifactorial intervention that includes pharmacological therapy along with behavioral modification and aspirin therapy decreased the risk of cardiovascular disease and the overall levels of glycosylated hemoglobin, blood pressure, and cholesterol and triglycerides levels as well as urinary albumin excretion

**Table 2:** Summary of participants' baseline characteristics in the reviewed studies.

Group	Look AHEAD trial		VADT		ACCORD trial		ADVANCE trial		UKPDS 38 trial	
	ILI	DSE	Standard therapy	Intensive therapy	Intensive therapy	Standard therapy	Intensive control	Standard control	Intensive therapy	Moderate therapy
Subjects (*n*)	2570	2575	899	892	5128	5123	5571	5569	758	390
Age (mean ± SD)	58.6 ± 6.8	58.9 ± 6.9	60.3 ± 9.0	60.5 ± 9.0	62.2 ± 6.8	62.2 ± 6.8	66 ± 6	66 ± 6	56.4 ± 8.1	56.5 ± 8.1
Male (*n*)	1044	1038	873	866	3143	3156	3195	3212	410	227
Female (*n*)	1526	1537	26	26	1985	1967	2376	2357	348	163
Average HbA1c (%)	7.25 ± 1.14	7.31 ± 1.20	9.4 ± 2.0	9.4 ± 2.0	8.3 ± 1.1	8.3 ± 1.1	7.51 ± 1.57	7.52 ± 1.54	6.9 ± 1.7	6.8 ± 1.5
Weight (mean ± SD)	F 94.8 ± 17.9 M 108.9 ± 19.0	F 95.4 ± 17.3 M 109.0 ± 18.0	214 ± 36	214 ± 36	93.5 ± 18.7	93.6 ± 18.7	78.2 ± 16.8	78.0 ± 16.8		
BMI (mean ± SD)	F 36.3 ± 6.2 M 35.3 ± 5.7	F 36.6 ± 6.0 M 35.1 ± 5.2	31.2 ± 4.0	31.3 ± 3.0	32.2 ± 5.5	32.2 ± 5.5	28 ± 5	28 ± 5	29.8 ± 5.5	29.3 ± 5.5
Systolic BP (mean ± SD)	128.1 ± 17.3	129.45 ± 17.1	132 ± 17	131 ± 17	136.2 ± 16.9	136.4 ± 17.2	145.0 ± 21.7	145.0 ± 21.4	159 ± 20	160 ± 18
Diastolic BP (mean ± SD)	69.69 ± 9.55	70.4 ± 9.72	76 ± 10	76 ± 10	74.8 ± 10.6	75.0 ± 10.7	80.8 ± 11.0	80.5 ± 10.8	94 ± 10	94 ± 9

ILI represents intensive lifestyle intervention; DSE represents diabetes support and education (conventional management); M and F represent males and females, respectively. Data are presented as (*n*) or mean ± standard deviation (SD).
